# Hypertriglyceridemic Waist Phenotype and Chronic Kidney Disease in a Chinese Population Aged 40 Years and Older

**DOI:** 10.1371/journal.pone.0092322

**Published:** 2014-03-24

**Authors:** Yongqiang Li, Chaomin Zhou, Xiaofei Shao, Xinyu Liu, Jia Guo, Ying Zhang, Honglei Wang, Xiaohong Wang, Bin Li, Kangping Deng, Qin Liu, Harry Holthöfer, Hequn Zou

**Affiliations:** 1 Department of Nephrology, Third Affiliated Hospital of Southern Medical University, Guangzhou, Guangdong, China; 2 National Centre for Sensor Research/BioAnalytical Sciences, Dublin City University, Dublin, Ireland; University of Sao Paulo, Brazil

## Abstract

**Objective:**

To examine the relationship between the HW phenotype and risk for CKD in a community population aged 40 years and older.

**Methods:**

A cross-sectional study was conducted in Zhuhai from June to October 2012. The participants were divided into three groups: Group 1, Waist circumference >90 cm in men or >85 cm in women and triglycerides ≥2 mmol/l; Group 3, Waist circumference ≤90 cm in men or ≤85 cm in women and triglycerides <2 mmol/l; Group 2, The remaining participants. The prevalence of the three subgroups and CKD were determined. The association between HW phenotype and CKD was then analyzed using SPSS (version 13.0).

**Results:**

After adjusting for age and sex, Group 1 was associated with CKD (OR 3.08, 95% CI 2.01, 4.73, P<0.001), when compared with Group 3. Further adjustment for factors which were potential confounders and unlikely to be in the causal pathway between the HW phenotype and CKD, Group 1 was still significantly associated with CKD. The OR for CKD was 2.65 (95% CI 1.65, 4.26, P<0.001). When adjusted for diabetes and hypertension, the association of Group 1 and CKD was still significant (OR 2.09, 95% CI 1.26, 3.45, P = 0.004). Group 2 was associated with CKD (OR 1.81, 95% CI 1.29, 2.53, P = 0.001), when compared with Group 3. Further adjustment for factors which were potential confounders, Group 2 was still significantly associated with CKD. The OR for CKD was 1.75 (95% CI 1.22, 2.51, P = 0.002). When adjusted for diabetes and hypertension, the association between Group 2 and CKD still existed. The OR for CKD was 1.48 (95% CI 1.01, 2.16, P = 0.046).

**Conclusion:**

Our results showed that HW phenotype was associated with CKD in the population aged 40 years and older.

## Introduction

Chronic kidney disease (CKD) has become a worldwide public health concern with an increasing prevalence during the last decade in China [1]. Zhang et al [1] recently reported that the overall prevalence of CKD in China was 10.8%. Outcomes of CKD include progression to end-stage renal disease (ESRD) and lots of complications of reduced kidney function such as increased cardiovascular events and a loss of disability-adjusted life years [2], [3]. CKD has become a big challenge to the health care system. The major renal replacement therapy is hemodialysis in China [4] with poor outcomes and high cost. In 2002, the annual incidence of hemodialysis in Beijing and Shanghai was 146.4 and 148.1 per million population, respectively [5]. The cost of renal dialysis alone is $14,300 per patient-year in China while the percapita disposable income was $1,210 in urban areas and $375 in rural areas [5]. It's a great burden to patients, society and country. The exorbitant cost of CKD, and its high mortality rate call for early detection and prevention of CKD. Pre-CKD has however obtained an increasing amount of attention [6]. Identifying high-risk subjects of CKD and taking appropriate intervention measures are indispensable.

The hypertriglyceridemic waist phenotype (HW phenotype) has obtained attention increasingly [7], [8]. Isabelle Lemieux and his colleagues firstly proposed the HW phenotype in 2000 [9]. The HW phenotype was defined as an elevated waist circumference (>90 cm in men and >85 cm in women), along with an elevated plasma triglyceride concentration (>2.0 mmol/L (177 mg/dl). Several studies have shown that the HW phenotype is a simple but sensitive marker of cardiovascular risk [9], [10]. A recent study suggestes that the HW phenotype is a better predictor of cardiovascular disease than the metabolic syndrome in non-diabetic subjects [11]. A recent study showed that the HW phenotype is associated with worse carotid atherosclerosis in CKD patients [12].

To the best of our knowledge, the association between the HW phenotype and CKD has never been evaluated in a developing country such as China.To fill this gap in the research field, we conducted this cross-sectional study of community population aged 40 years and older to examin the relationship between the HW phenotype and CKD in the city of Zhuhai in Guangdong province, China. Data were collected from1753 community residents older than 40 years from June to October, 2012. Participants were selected using a multi-stage stratified random cluster sampling method.

## Methods

### Subjects

The Ethics Committee of The Third Affiliated Hospital of Southern Medical University, Guangzhou, approved this study. This study was performed fulfilling the principles of Helsinki Declaration and the International Guidelines for Ethical Review for Epidemiological Studies. Data was collected from1753 community residents older than 40 years from June to October, 2012. Participants were selected using a multi-stage stratified random cluster sampling method. Step 1, two communities were selected randomly from Wanzhai Town; step 2, in each of the two selected communities, 500 families were randomly sampled as the target family; and step 3, all the residents aged 40 years and older in the selected families were sampled. The exclusion criteria included: missing gender; age; education status; missing any item of lifestyle information (for example, smoking status, alcohol intake, and physical activity); not being in the fasting state for at least 10 hours; missing any item of waist measurement, blood pressure (BP), body mass index (BMI), blood glucose, serum high-density lipoprotein (HDL) cholesterol, and triglyceride (TG) levels information. Using this method, a total of 1753 participants from 2198 residents completed the survey, with a response rate of 79.8%. 173 participants were assigned to Group 1.541 participants were assigned to Group 2 and 820 participants belonged to Group 3 according to their waist circumferences and triglyceride levels.

Participants were recruited by mail and home visits. First, we informed participants by mail. Then we visited the families and got the filled questionnaires from the participants. All participants signed a letter of informed consent.

### Data collection

Data on age, sex, personal history (coronary artery disease, hypertension, and diabetes) and details about lifestyle (current or past cigarette smoking, alcohol intake, diet habits, educational status, and physical activity) were obtained through questionnaires. The participants completed the questionnaires under the professional guidance of physicians, medical students or nurses. All of them had received intensive training.

### Physical measures

Physical measures were conducted in the community clinics. A well-trained nurse measured body weight, height, waist circumference and blood pressure in the morning between 08:00 am and 11:00 am. Body height and weight were measured with a calibrated clinical scale and stadiometer. Body mass index (BMI) was calculated as weight (kg) divided by height squared (m2). Waist circumference was taken midway between the last rib and iliac crest with the participants standing with light garments and breathing out gently [14]. The waist circumference was measured two times and the average of two measures was recorded to the nearest 0.1 cm. Blood pressure was measured three times with a calibrated mercury sphygmomanometer and the average value was calculated. BP was measured in the sitting position after resting for at least 5 minutes.

### Laboratory values

Following an overnight fast of at least 10 hours, blood specimens were collected for plasma glucose, HDL cholesterol, LDL cholesterol, triglycerides, serum creatinine, highsensitivity C-reactive protein (CRP) and insulin. First morning urine samples were collected from all participants, except those women who were actively menstruating.

Individuals having urinary tract infection symptoms were also excluded from the urine test. All specimens were transported to the central laboratory in the Third Affiliated Hospital of Southern Medical University within 3 hours from collection sites and stored at 4°C until analysis. Methods we used to detect laboratory variables had been described in our previous paper [13].

### Definition of HW phenotype

The HW phenotype was defined as elevated waist circumference (>90 cm in men and >85 cm in women), along with an elevated plasma triglyceride concentration (>2.0 mmol/L (177 mg/dl) [12].

### Determination of CKD

The estimated glomerular filtration rate (eGFR) was estimated using a formula from the Chinese-Modification of Diet Renal Disease (C-MDRD) study: GFR (ml/min/1.73 m^2^) = 175× (Scr)^−1.234^× (Age)^−0.179^×(if female, ×0.79) [15]. Reduced renal function was defined as an eGFR of less than 60 mL/min per 1.73 m^2^. For practical purposes, albuminuria was defined as a spot urinary albumin-to-creatinine ratio (ACR) higher than 30 mg/g. CKD was defined as an eGFR of less than 60 ml/min per 1.73 m^2^ or albuminuria.

### Determination of Diabetes and Hypertension

A blood pressure (BP) of 130/85 mmHg or higher or receiving treatment for previously diagnosed for hypertension. A fasting blood glucose (FBG) of 5.6 mmol/l or higher or with previously diagnosed for type 2 diabetes.

#### Socioeconomic factors

The socioeconomic factors used in this study were defined on educational status. Education status was classified into three categories: (1) 0 years of schooling;(2) primary school or junior middle school; (3) high school or above.

#### Health behavior factors

Alcohol consumption was evaluated based on the frequency of alcohol intake as recorded on the health interview questionnaire. We divided the residents into three groups: (1) No drinking; (2) Current alcohol use; (3) former but no present drinking. For physical exercise, the participants were divided into two groups: (1) no physical activity; (2) physical activity. For smoking, they were divided into three groups: (1) former smokers who quit smoking prior to the survey; (2) current smoker; (3) non-smoker.

### Statistical Analysis

Data were analyzed using SPSS (version 13.0). Continuous variables were shown as mean ± standard deviation if they had normal distribution. Median and interquartile range were used to show skewed distributed continuous variables. The categorical variables were presented as absolute and relative (%) values or proportion. A two-tailed p value <0.05 was considered significant.

We divided the participants into three groups according to their waist circumferences and triglyceride levels: Group 1, waist circumference >90 cm in men or >85 cm in women and triglycerides ≥2 mmol/l; Group 2, waist circumference <90 cm in men or <85 cm in women along with a plasma triglyceride concentration of ≧2.0 mmol/L/waist circumference ≧90 cm in men or ≧85 cm in women along with a plasma triglyceride concentration of <2.0 mmol/L; Group 3, waist circumference ≤90 cm in men or ≤85 cm in women and triglycerides <2 mmol/l.

Baseline characteristics within Group 1, Group 2 and Group 3 were examined using the chi-squared test for categorical variables and one-way ANOVA or Wilcoxon rank-sum test for continuous variables.

Mutiple logistic regression models were used to explore whether the HW phenotype is associated with CKD. The first model was adjusted for age and sex. Next, history of hypertension, history of coronary heart disease, history of stroke, history of malignancy, current smoker, current alcohol use, physical inactivity and education status were added into the model. Furthermore we examined whether the associations were independent of diabetes or hypertension. Diabetes and hypertension were added to the above covariates. Group 3 was considered as the reference group.

## Results

Initially there were 1753 participants aged 40 years and older in our study and all participants were Han ethnic. 219 subjects were excluded because of missing data for serum creatinine, ACR, triglyceride or waist circumference. Finally, we included 1534 participants with mean age 57.21±10.97 years in the current study. 173 participants were assigned to Group 1. 541 participants were assigned to Group 2 and 820 participants belonged to Group 3 according to their waist circumferences and triglyceride levels.

### Baseline characteristics of the participants based on HW phenotype

As shown in Table 1, patients in Group 1 had significantly higher serum uric acid, serum C-reactive protein, HOMA-index, BMI than those in Group 2 and Group 3 (P<0.001). Additionally, these values in Group 2 were significantly higher than those participants in Group 3 (P<0.001). Participants in Group 1 were older and had a higher prevalence of hypertension, diabetes, a higher diastolic blood pressure, higher levels of fasting glucose, ACR, and lower serum high-density lipoprotein than those in Group 2 and Group 3 (P<0.001). Current smoker status and current alcohol use statuses are more common in Group 1 than those in groups 2 and 3. There were no differences in educational status (high school or above) and history of coronary heart disease among the participants in the three groups.

**Table 1:** Baseline characteristics of the participants based on HW phenotype.

	Group1	Group2	Group3	
	n = 173	n = 541	n = 820	p
Age (years)	59.24±11.44	58.25±10.34	56.1±11.16	<0.001
Male (%)	86(49.7)	208(38.4)	274(33.4)	<0.001
**Clinical Characteristics**				
Hypertension history (%)	77 (44.5)	156 (28.8)	138 (16.8)	<0.001
Diabetes history (%)	19 (11.0)	51(9.4)	46(5.6)	0.007
Coronary heart disease history (%)	5 (2.9)	14 (2.6)	28 (3.4)	0.68
Current smoker (%)	33 (19.1)	61 (11.3)	91 (11.1)	0.011
Current alcohol use (%)	52 (30.1)	124 (22.9)	156 (19.0)	0.004
Educational status (≥high school)(%)	51 (29.5)	150(27.7)	269(32.8)	0.13
Systolic blood pressure (mm Hg)	137.15 ±18.27	135.37±18.48	126.63±19.71	<0.001
Diastolic blood pressure (mm Hg)	84.16±9.44	81.44±10.38	76.52±10.215	<0.001
Body mass index (kg/m 2)	26.63±2.69	25.23±3.07	21.86±2.65	<0.001
Waist circumference (cm)	95.57±5.91	90.38±8.98	77.79±6.55	<0.001
**Laboratory values**				
Fasting glucose (mmo/l)	5.58±1.58	5.31±1.37	4.88±0.96	<0.001
Serum C-reactive protein (mg/l)	95.57±5.91	90.38±8.98	77.79±6.55	<0.001
Serum triglyceride (mmol/L)	2.5 (2.32–3.33)	1.53 (1.13–2.26)	1.04 (0.78–1.33)	<0.001
Serum low density lipoprotein (mmol/l)	3.13±1.01	3.37±0.93	3.26±0.84	0.005
Serum high density lipoprotein (mmol/l)	1.38±0.28	1.45±0.28	1.63±0.34	<0.001
Very low density lipoprotein (mmol/l)	1.32±0.45	0.81±0.43	0.49±0.17	<0.001
HOMA-index (uU/ml.mmol/ml)	3.2 (2.34–4.64)	2.43 (1.69–3.45)	1.38 (0.99–1.93)	<0.001
Serum creatitine (umol/L)	80.84±17.38	75.03±18.46	71.81±15.87	<0.001
eGFR (mL/min/1.73 m 2)	88.27±16.37	95.17±20.91	99.34±21.67	<0.001
ACR (mg/g)	12 (6–23)	9 (6–17)	7 (5–13)	<0.001
Serum uric acid (umol/L)	421.12±97.47	375.09±97.91	325.46±86.14	<0.001

Note: Values expressed as absolute and relative (%) percent for categorical variables and mean ± SD for continuous variables. Note: Values expressed as absolute and relative (%) percent for categorical variables and Mean ± SD or median (25th to 75th percentiles) for continuous variables.

Abbreviations: ACR, albumin-creatinine ratio; eGFR: estimated Glomerular filtration rate; HOMA-IR: Homeostatic model assessment of insulin resistance;

### Prevalence of CKD in the three subgroups

**Figure 1:**
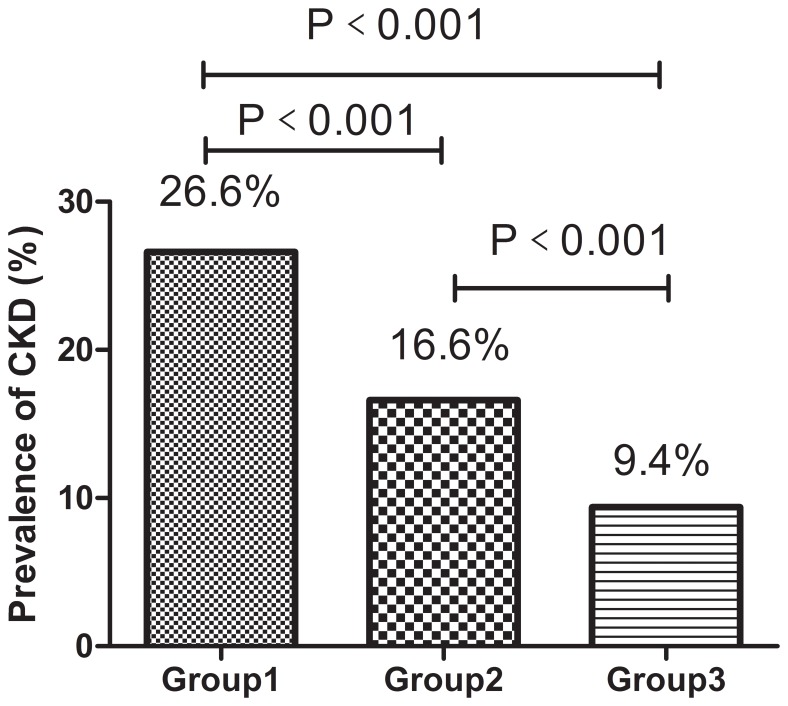
Prevalence of CKD in 1534 subjects based on HW phenotype.

As shown in Fig. 1, there were 46/173 subjects (26.6%) with CKD in group 1. Participants in group 2 had a higher prevalence of CKD 90/541 (16.6%) than those in group 3, 77/820(9.4%) (p<0.001). Participants in group 1 had the highest prevalence of CKD among the three subgroups. (p<0.001).

### Association of the HW phenotype with CKD

As shown in Table 2, Group 1 was associated with CKD (OR 3.08, 95% CI 2.01, 4.73, P<0.001) in model one, when compared with Group 3. Further adjustment for factors which were potential confounders and unlikely to be in the causal pathway between the HW phenotype and CKD had an impact on the odd ratios, Group 1 was still significantly associated with CKD. The odd ratio for CKD was 2.65 (95% CI 1.65, 4.26, P<0.001). When adjusted for diabetes and hypertension, the association of Group 1 and CKD was still significant (OR 2.09, 95% CI 1.26, 3.45, P = 0.004). Group 2 was associated with CKD (OR 1.81, 95% CI 1.29, 2.53, P = 0.001) in model one, when compared with Group 3. Further adjustment for history of hypertension, history of coronary heart disease, history of stroke, history of malignancy, current smoker, current alcohol use, physical inactivity, educational status, Group 2 was still significantly associated with CKD. The odd ratio for CKD was 1.75 (95% CI 1.22, 2.51, P = 0.002). When adjusted for diabetes and hypertension, the association between Group 2 and CKD still existed. The odd ratio for CKD was 1.48 (95% CI 1.01, 2.16, P = 0.046).

**Table 2:** Association of the hypertriglyceridemic waist phenotype with CKD.

	Model one^a^		Model two^b^		Model three ^c^	
	OR (95% CI)	P value	OR (95% CI)	P value	OR (95% CI)	P value
Group 3	**Reference**		**Reference**		**Reference**	
Group 1	3.08(2.01–4.73)	<0.001	2.65(1.65–4.26)	<0.001	2.09(1.26–3.45)	0.004
Group 2	1.81(1.29–2.53)	0.001	1.75(1.22–2.51)	0.002	1.48(1.01–2.16)	0.046

a.Adjusted for age, sex.

b.Adjusted for age, sex, history of hypertension, history of coronary heart disease, history of stroke, history of malignancy, current smoker, current alcohol use, physical inactivity, educational status.

c. Adjusted for above + diabetes and hypertension.

## Discussion

In the present study, we found that population aged 40 years and older characterized by the presence of the HW phenotype were at a higher risk of CKD than those without this phenotype. The HW phenotype was positively associated with CKD. Meanwhile, This relationship was independent of age, sex, history of hypertension, history of coronary heart disease, history of stroke, history of malignancy, current smoker, current alcohol use, physical inactivity, educational status, diabetes and hypertension.

During recent years the HW phenotype has obtained an increasing amount of attention. Accumulating evidences show that the HW phenotype might be a simple, yet a significant marker of CAD [9], [16]. The HW phenotype was associated with worse carotid atherosclerosis in CKD patients [12] and was positively associated with hyperglycemia [17]. To the best of our knowledge, this is the first study evaluating the relationship between the HW phenotype and CKD. In the present study, individuals with the HW phenotype were 2.09-fold as likely to have CKD as were those with both low waist circumference and low TG concentration.

Multiple potential mechanisms might be responsible for a higher risk of CKD in individuals showing a HW phenotype even after adjustment for life style, hypertension, diabetes and other potentially confounding factors. The HW phenotype is actually a marker of visceral obesity [18]. Visceral obesity or excess fatty acids accompanied with increased levels of triglyceride may result in accumulation of fat at ectopic tissues such as liver, pancreatic b-cells and the kidneys [11], [19]. The ectopic accumulation of fat at these organs would result in steatohepatitis, insulin resistance, compression of the kidneys and therefore hypertension, diabetes, hyperuricemia and unfavorable renal hemodynamic pattern [20], which contribute to CKD. In another hand, there is a close correlation between hypertriglyceridemia and uric acid (UA) production. Production of UA in viscerally obesity subjects is higher [21]. Furthermore, the hypertriglyceridemic waist is reported to be an important factor increasing high-sensitivity C-reactive protein levels [22]. Both hyperuricemia and high levels of C-reactive protein contribute to CKD. Our results that participants with the HW phenotype had higher levels of fasting glucose, serum uric acid, VLDL-C, insulin, blood pressure and C-reactive protein and therfore higher risk of CKD might further support these mechanisms. These results were consistent with previous reports [9], [23]–[25].

The increasing prevalence of CKD requests us to find out more efficient markers for monitoring the prevalence of CKD. Several studies have suggested that obesity and the metabolic syndrome are independent predictors of CKD [17], [26]–[29]. Body mass index (BMI) was widely used as a marker of obesity. But fluid overload and body fat distribution should be considered. In the present study, participants in all groups were non-obese according to their respective BMI. But they were at a high risk of CKD especially in those with the HW phenotype, which is a marker of visceral obesity. Chinese are known to have a predisposition to visceral fat accumulation despite having generally low BMI [30]. These results supported that BMI was not an ideal marker of obesity and was not as sensitive as the HW phenotype in capturing the risk of CKD. Though waist circumference is recommended to be a relatively ideal marker of obesity [31], it is rather difficult to identify subcutaneous and visceral adiposity. Several studies indicate that it is visceral obesity but not the subcutaneous adiposity that correlates with the metabolic abnormalities [32], [33] and not all population with an elevated waist circumference are viscerally obese. Hence the waist circumference alone is not an ideal marker of obesity. Recent studies showed that the HW phenotype is a better marker of visceral obesity [18].

The metabolic syndrome and its components were also reported to be risk factors of CKD [34]. However, recent studies suggest that the HW phenotype predicts metabolic abnormalities better than the metabolic syndrome [9]. In Henry S Kahn's study, enlarged waist with elevated triacylglycerols alone identified more persons with greater concentrations of LDL cholesterol and apolipoprotein B than did metabolic syndrome alone [35]. In addition, the diagnosis of metabolic syndrome needs more laboratory data and therefore it's somewhat easier to evaluate the HW phenotype. Based on those advantages of the HW phenotype and the finding of our study that the HW phenotype was significantly associated with CKD, we advise that the HW phenotype might be a simple but sensitive marker for screening individuals who have higher CKD risk factors.

There are several limitations in the current study. Firstly, the cross-sectional nature of our study disabled us to make causal inferences. Prospective studies are needed to prove whether this phenotype could predict major outcomes such as CKD events or mortality. Secondly, all the indicators of CKD (eGFR and ACR) were obtained on the basis of single measurements without repeating tests. Thirdly, Cut-offs of 90 cm and 2.0 mmol/L in men of 85 cm and 2.0 mmol/L in women were advocated for the diagnosis of visceral obesity and metabolic syndrome in Europeans, these cut-offs need to be validated for Chinese population, in different Chinese ethnic groups, genders and across different age groups. Fourthly, Participants were selected from the chosen families. Lifestyle and even potential genetic determinants might widely differ between participants from our selected families and non-related individuals from the general population, these issues might have a potential effect on the incidence of CKD in our study.

In summary, our study showed that the HW phenotype was associated with the presence of CKD in the population aged 40 years and older, from which we can speculate that the HW phenotype might be considered as a simple, yet sensitive marker for identifying adults at risk of CKD in the population aged 40 years and older.
